# Synovial sarcomas: A single surgeon experience of 130 cases

**DOI:** 10.1002/jso.26976

**Published:** 2022-06-07

**Authors:** Lorenzo Andreani, Edoardo Ipponi, Olimpia Mani, Ginevra Bayon, Alfio Damiano Ruinato, Fabio Cosseddu, Antonio D'Arienzo, Rodolfo Capanna

**Affiliations:** ^1^ Department of Orthopaedics and Trauma Surgery University of Pisa Pisa Italy

**Keywords:** oncological surgery, survival, synovial sarcoma, tumor resection

## Abstract

**Background:**

Synovial sarcoma is a rare malignant tumor that generally requires a multidisciplinary therapeutic approach. In this study we report the experience of a single surgeon, evaluating surgical and oncological outcomes of the cases he treated through his 30 years carrier.

**Methods:**

We enrolled patients treated surgically between 1988 and 2018. Surgical and medical treatments, as well as surgical and oncological results, were investigated.

**Results:**

One hundred and thirty cases were included. Surgical resection was carried out achieving wide margins in 90% of the cases. At their latest follow‐up, 76 patients were continuously disease free, 16 were no evidence of disease, and other 16 were alive with disease. Twenty cases were dead of disease and two dead of other causes. Twenty‐five patients (19%) had local recurrence of synovial sarcoma through their postoperative intercourse. Thirty‐seven patients (28%) were diagnosed with at least a metastasis during their follow‐up. The global survival of our population, at each patient's latest follow‐up, was 82%. Cases with tumor size above 5 cm had a significantly higher risk to develop metastasis (*p* = 0.002).

**Conclusions:**

Synovial sarcoma is a threatening disease and represents a challenge for oncological physicians and surgeons. Early diagnosis and multidisciplinary approach are mandatory to limit the spread of synovial sarcomas, maximizing the effectiveness of surgery and the other treatments.

## 1 INTRODUCTION

Synovial Sarcoma is a rare tumor that alone accounts for 5%–10% of all soft tissue sarcomas. Its incidence is 1–3 cases per million per year in the general population.^1^ Although unrelated to the synovium, synovial sarcoma was originally named because of its histologic resemblance to synovial cells.^2^

It occurs mostly in adolescents and young adults and is typically located in the lower limb.^3^ Although etiology is commonly believed to be multifactorial, more than 90% of synovial sarcomas have a characteristic *t*(X,18) translocation (p11.2;q11.2), that causes the fusion of the *SS18* gene on chromosome 18 with one of three closely related genes (*SSX1*, *SSX2*, and *SSX4*) on the X chromosome, resulting in an aberrant *SSX* transcription.^3^, ^4^, ^5^, ^6^ This genetic anomaly unites the transcriptional activating domain of *SS18* and the transcriptional repressor domains of *SSX*, giving birth to a fusion product that is supposed to play an important role in the pathogenesis of Synovial Sarcoma, although the exact mechanism still remains unclear.

These lesions commonly have a quick and aggressive growth with tendency to develop metastasis in anatomical sites distant from the one of origin.

Several studies testified to high mortality and low survival rates; hence synovial sarcoma is generally considered a high‐grade tumor with a poor prognosis and must be treated as such.^2^, ^7^, ^8^

The complexity of the disease makes it necessary to use a multidisciplinary approach with combinations of chemotherapy, surgery, and radiant therapy. Systemic treatment is nowadays carried out with Ifosfamide‐based regimens to which the tumor is sensitive.^2^, ^9^, ^10^ Local treatment instead mainly relies on surgery to eradicate the tumoral mass and potentially solve the disease in case metastasis is absent at the moment of the intervention. Perioperative radiant therapy, for its part, seems to play a role in the increment of postoperative survival rate.^11^

A histologic subtype, size, localization, and metastasis at presentation, along surgical margins, are amongst the most reasonable prognostic factors described in the literature so far.^12^

The operative approach to synovial sarcomas is particularly challenging for oncologic surgeons. Due to their high malignancy, resection should dovetail the respect of the nearby healthy tissues and the achievement of wide margins, necessary to minimize the risk of local recurrence, and we report our experience with 130 cases of synovial sarcoma treated with surgery between 1988 and 2018. In our study, we assessed oncologic outcomes and complications rates of each patient, evaluating possible statistical associations between supposed prognostic factors and postoperative survival.

## 2 MATERIALS AND METHODS

Our study consisted of a retrospective review of all patients with synovial sarcoma who were treated with surgery by Professor RC and his equips between 1988 and 2018.

**Figure 1:**
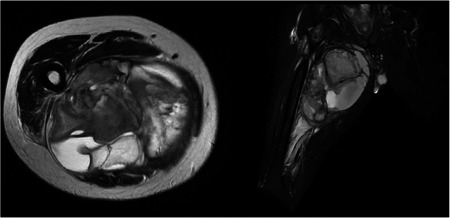
Magnetic resonance images of a wide synovial sarcoma localized in the thigh.

For each patient, we collected data regarding their age and gender, and previous pathological anamnesis. For each tumor, we recorded site and size, estimated using preoperative magnetic resonance imaging and computed tomography scan images (Figure 1). The same images, alongside nuclear medicine investigations (scintigraphy and/or FDG‐PET) were used to stage the disease and assess its eventual spread in distant locations. Once tumor resection was carried out, the resected masses underwent histopathological analysis to establish a definitive diagnosis of synovial sarcoma. Microscopical examinations were also used to evaluate surgical margins of resection—expressed as wide, marginal, or intralesional—and histological grading according to the FNCLCC classification. For each case, the recourse to chemotherapy or radiant therapy, whether they were adjuvant or neoadjuvant, was considered and recorded. Each intra‐operative and postoperative complication, of grade II or higher according to the Clavien–Dindo classification, was included and mentioned in our study.

Postoperative follow‐up consisted of periodic orthopedic and oncologic office visits, with clinical and imaging evaluations. Metastasis, local recurrences, and deceases were recorded and oncological status was attributed to each case at their latest follow up, dividing them into continuously disease free (CDF), no evidence of disease (NED), alive with disease (AWD), dead of disease (DOD), and dead of other causes (DOC).

### 2.1 Statistics

Statistical analysis was performed using Stata SE 13 (StataCorp LLC). Statistical significance was set at 0.05 for all endpoints.

## 3 RESULTS

Our population consisted of 130 patients: 68 females and 62 males. Their mean age at surgery was 45.5 years (10–89) and 13 of them (10%) were under 18 years of age. The average tumor size, expressed as a greater diameter, was 6.1 cm (3–18 cm). The neoplastic mass was 5 cm or shorter in 70 cases (54%), between 5 cm and 10 cm in 42 cases (32%), and over 10 cm in the remaining 18 cases (14%). Resected tumors involved the lower limb in 93 patients (72%), the upper limb in 33 (25%), and the trunk in the remaining 4 (3%).

Sixty‐four of our 130 patients (49%) received radiant therapy through their preoperative and/or postoperative intercourse. Radiant therapy was administered as a neoadjuvant treatment in 27 cases (21%) and as an adjuvant in 40 cases (31%).

Fifty‐eight of our 130 patients (45%) received chemotherapy. The treatment was administered before surgery in 34 cases (26%) and after surgery in 38 cases (31%).

Surgical resection was carried out achieving wide margins in 117 cases (90%). The remaining 13 cases had marginal (5; 4%) or intralesional resections (8; 6%).

At their latest follow‐up, 76 patients (58%) did never show any sign of local recurrence or metastasis (CDF). New presentations of disease were diagnosed after surgery in the remaining 54 patients (42%). Among them, 16 cases that underwent further surgical interventions which successfully eradicated the tumor and are therefore to be considered with no evidence of disease (NED). Over 16 cases (12%) are still alive, despite the diagnosis of a secondary lesion (AWD). The remaining 22 patients died in the months and years that followed surgery: 20 died of disease (DOD), whereas two died of other causes (DOC).

**Figure 2:**
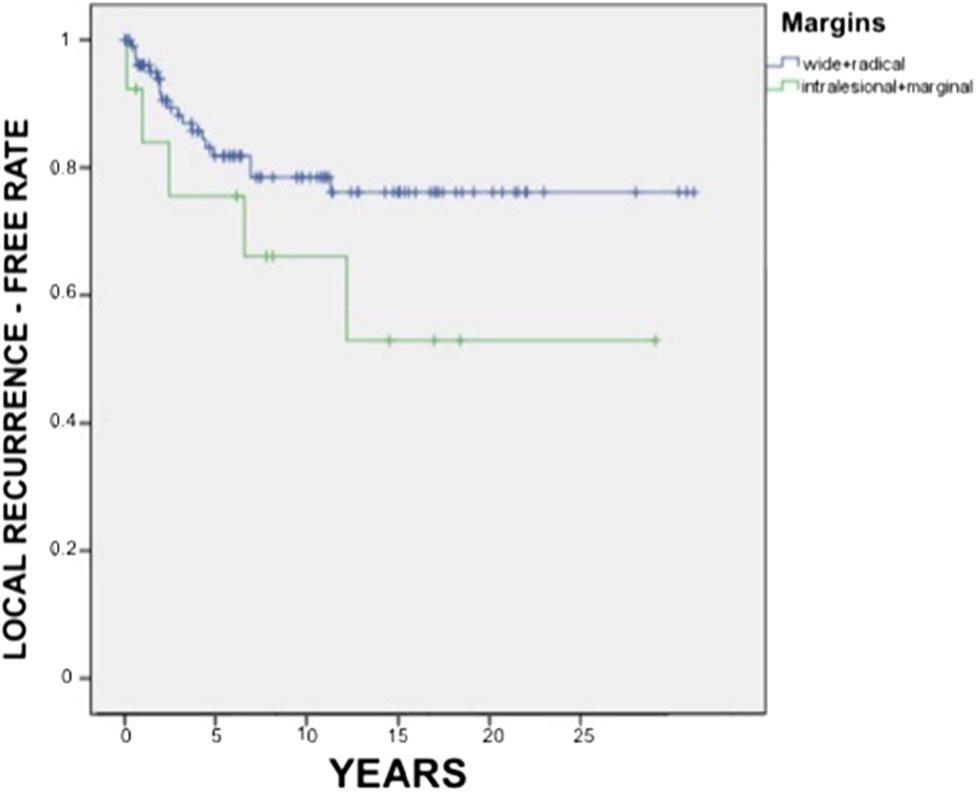
Local recurrence‐free over time according to whether the excision was marginal or wide.

Twenty‐five patients (19%) had local recurrence of synovial sarcoma through their postoperative intercourse. The percentage of local recurrence was 2% after 1 year, 5% after 2 years, 8% after 5 years, and 14% after 10 years. Cases that had marginal resection had a local recurrence incidence of 31%, whereas in those who had radical interventions or wide margins the percentage decreased to 18% (Figure 2). Despite this difference, this result was not statistically significant according to a Fisher Exact Test (*p* = 0.274). Similar findings were obtained for the relation between preoperative radiant therapy and local recurrence of synovial sarcoma. Although the prevalence of postoperative local recurrence was lower in cases who underwent neoadjuvant radiant therapy (16%) compared with the ones who did not receive the treatment (22%), the size of our population did not furnish enough statistical significance to suggest a strong link between radiations and recurrence risk (*p* = 0.595 according to a Fisher Exact Test).

Thirty‐seven patients (28%) were diagnosed with at least a metastasis during their follow‐up. Metastasis‐free survival was 95%, 87%, 76%, and 73%, respectively, 1 years, 2 years, 5 years, and 10 years after surgery.

Those who received postoperative chemotherapy had a risk of 26% to develop postoperative metastasis; the rest of our population had a risk of 29%. Kaplan–Meier statistics and log rank tests did not report any significant correlation between the treatment or localization and the survival without local recurrence or distant metastasis. We also compared the rates of local recurrence and systemic metastasis dividing our population based on tumor size. Cases that had a lesion larger than 5 cm had higher rates of postoperative local recurrence and metastasis: 17% versus 22% and 17% versus 42%, respectively. Although *χ* ^2^ tests did not provide sufficient evidence to assess size as a risk factor for local recurrence, the same test reported that cases with tumor size above 5 cm had a significantly higher risk to develop metastasis (*p* = 0.002).

Overall survival was 97% 1 year after surgery, 93% after 2 years, 89% after 5 years, and 85% after 10 years. The global survival of our population, at each patient's latest follow‐up, was 82%.

## 4 DISCUSSION

The synovial sarcoma is a rare and extremely aggressive soft tissue tumor. Although adjuvant and neo‐adjuvant treatments can be effective in selected patients with an ancillary role, to this date surgery still represents the only therapeutic approach capable of eradicating the disease.^13^, ^14^, ^15^ Today the mainstay of treatment consists of surgical excision with wide margins, to minimize the risk of local recurrence. At the down of orthopedic oncology, the surgery of choice for sarcomas involving the upper or lower limbs was represented by the amputation of the affected body segment.^16^ Since the 1970s the advances in surgical technologies, associated with the blooming of reconstructive approaches and the development of both chemotherapy and radiant therapy led to a new era in surgical oncology for sarcomas of the extremities: the limb‐sparing surgery. Unlike previous radical approaches, limb sparing surgery allows the excision without sacrificing the involved limb, performing a reconstruction of the removed bone or soft tissues when necessary.^17^ To this day amputations are reserved only for a limited share of cases. Limb sacrifice should still be considered for those cases in which the tumor location inevitably calls up to the damage of vital structures, implying poor limb function.^16^ Similarly, older patients or those with extensive medical comorbidities may not be able to tolerate a major operation, and amputation can be considered.^16^ In the remaining cases limb sparing surgery represents the best operative option, dovetailing the pursuit of postoperative functionality with the necessity to achieve wide resection margins. As emerges from previous literature, the quality of surgical resection margins plays a crucial role in preventing local recurrence and therefore maximize oncological outcome in terms of overall survival.^15^, ^18^, ^19^, ^20^ Although recommendations regarding margin management should be taken into context of the individual patient, position and size should be considered while planning each patient's postoperative treatment. As emerged from previous studies, for superficial tumors or small (<5 cm) deep tumors not intimately associated with critical structures, wide excision with negative margins alone could be considered sufficient.^15^, ^20^, ^21^, ^22^ In the case of marginal resection, instead, adjuvant radiant therapy should be considered to reduce the risk of local recurrence of the disease.^15^, ^20^, ^21^, ^22^, ^23^, ^24^, ^25^, ^26^

Advances in orthopedic surgery, radiation therapy, and oncology, in association with a better comprehension of the tumor both on a histopathological and a radiographical point of view significantly increased the overall survival of patients who are diagnosed with synovial sarcoma. In fact, while early cohorts from the 1960s had a 25%–51% 5‐year survival rate, the percentage has been progressively increasing through the following decades. The current literature suggests that the 5‐year survival rates are now ranged between 59% and 75%.^3^, ^15^, ^27^, ^28^, ^29^, ^30^ In our population the survival rate 5 years after surgery amounts to 89%.

Although the fact that this outcome falls beyond the aforementioned range could be at least in part attributable to the differences of populations examined, the rate obtained in our series is encouraging and continues the trend of a progressive increment of life expectancy con synovial sarcomas that are emerging in latest literature.^3^, ^21^, ^30^ This result testifies the effectiveness of a good multidisciplinary treatment, in which patients are treated not only by surgeons or oncologists alone but by an alliance of different medical figures—including orthopedics, medical oncologists, radiotherapists but also pathologists, radiologists, and specialists in nuclear medicine—gathered in highly specialized cancer centers.

In 2011 Krieg et al.,^31^ testified that synovial sarcoma tends to recur even several years after surgical eradication of the primary tumor, later compared with the vast majority of other malignant tumors, including many different sarcomas. Despite the lower incidence of secondary lesions, our population confirms the same tendency, suggesting that the risk of local recurrences and metastasis decreases slightly and in a large time span.

In our population, cases with tumor size higher than 5 cm had poorer survival rates, associated with a higher rate of local recurrences and—in particular—metastatic lesions. Lesions larger than 5 cm had a 45% risk of developing metastases and the rate rises to 60% for cases with a diameter above 10 cm. The risk in those sub‐populations is significantly higher than the rate of 17% we had in cases with tumors smaller than 5 cm. These findings are confident with the ones reported by Naing et al. in 2014,^11^ who demonstrated that size predicted worse overall survival.

No significant correlation between the administration of radiant therapy, whether it was performed pre‐operatively or postoperatively, and oncological outcomes emerged from our data.

We are conscious our study is not free of limitations. One of them is represented by the retrospective nature of our study, which did not allow the complete standardization of the postoperative follow‐up procedures for each patient. Another limitation is represented by the wide time span covered by our study. Between 1988 and 2018, surgical technologies developed, as well as chemotherapy, radiation therapy, and imaging technologies which had impressive innovations through those 30 years. These changes inevitably reduced the grade of standardization in our cohort.

Despite these limitations, our study includes a relatively large number of patients treated with surgery due to an extremely rare disease like synovial sarcoma. We gathered all the cases treated by the same surgeon and his teams through 30 years of activity, innovation, and research.

## 5 CONCLUSION

Our outcomes suggest that an early diagnosis and prompt therapies are mandatory to limit the local growth and the systemic spread of synovial sarcomas, thereby maximizing the effectiveness of surgery and other treatments. Orthopedic surgery, especially if performed in highly specialized centers in association with collegial evaluations and multidisciplinary therapeutic approaches, can be effective and provide good oncological outcomes, and encouraging survival rates for the years that follow the treatment.

## CONFLICT OF INTEREST

The authors declare no conflict of interest.

## SYNOPSIS

This study evaluates the oncological outcomes of 130 patients with synovial sarcoma treated with surgical resection over three decades. At their latest follow‐up, 25 patients (19%) had local recurrence and 37 patients were diagnosed with metastasis after surgery. Seventy‐six patients were CDF, 16 NED, 16 AWD, 20 DOD, and 2 DOC.

## Data Availability

The data that support the findings of this study are available from the corresponding author upon reasonable request.
